# Usefulness of the Cognitive Composition Test as an Early Discriminator of Mild Cognitive Impairment

**DOI:** 10.3390/jcm12031203

**Published:** 2023-02-02

**Authors:** Yoshiki Tamaru, Hiroyuki Sumino, Akiyoshi Matsugi

**Affiliations:** 1 Faculty of Rehabilitation, Shijonawate-Gakuen University, Hojo 5-11-10, Daito 574-0011, Osaka, Japan; 2 Department of Rehabilitation, Geriatric Health Service Facility Sungarden Fuchu, Sansocho, 2-1-15, Izumi 594-0021, Osaka, Japan

**Keywords:** mild cognitive impairment, Montreal cognitive assessment, cognitive composition test, mini mental state examination, trail making test

## Abstract

Mild cognitive impairment (MCI) is the preliminary stage of dementia, which is a serious social problem worldwide. This study aimed to investigate whether the Cognitive Composition Test (CCT) is effective for the early diagnosis of MCI. A total of 104 older adults underwent the Montreal Cognitive Assessment (MoCA), the Mini-Mental State Examination (MMSE), the Trail Making Test Parts A (TMT-A) and B (TMT-B), and our newly prototyped cognitive composition test (CCT). We created three types of CCT (CCT-A, CCT-B, and CCT-C) with different degrees of difficulty. First, we examined the concurrent validity of CCT-A, CCT-B, and CCT-C with the MoCA, MMSE, TMT-A, and TMT-B. All participants were classified into the healthy control (HC) and MCI groups based on their scores in the Japanese versions of the MoCA and MMSE. The HC and MCI groups were compared using the TMT-A, TMT-B, CCT-A, CCT-B, and CCT-C. Finally, we examined the sensitivity for discrimination of CCT-C. CCT-C had a higher discrimination sensitivity than TMT-A, TMT-B, CCT-A, and CCT-B, with a cut-off value of 65.75 s, a sensitivity level of 0.844, and a specificity of 0.776. It may be a useful screening tool for the early diagnosis of the early-stages of dementia, such as MCI, in asymptomatic older adults.

## 1. Introduction

Japan has a higher aging rate than other countries; approximately 25.1% of Japan’s total population was over the age of 65 years in 2013. By 2035, one-third of the population in Japan is expected to be at least 65 years old [1]. Moreover, dementia, which increased in incidence with the aging of the population, has become a serious social problem both worldwide and in Japan. Mild cognitive impairment (MCI) is the preliminary stage of dementia.

The early diagnosis of MCI is crucial for the prevention of dementia, since appropriate treatment at this stage can delay or reduce the severity of dementia [2]. Between 10 and 20% of adults above the age of 65 years are diagnosed with MCI [3], and approximately 10% of adults with MCI progress to Alzheimer’s dementia (AD) [4]. Furthermore, older adults with MCI progress to clinically probable AD more rapidly than age-matched healthy older adults [5]. Manly et al. [6] reported that the recovery rates from MCI to a healthy stage ranged from 14% to 44%. In addition, Shimada et al. [2] conducted a 4-year follow-up study of older adults with MCI and reported that 46% of them may recover to a healthy state by repeating appropriate measures such as aerobic exercise and cognitive function training. This finding indicates that cognitive impairment can be prevented from progressing, and that patients can recover to a normal state in the early stages. Therefore, early detection at the MCI stage is important for treating this condition [7]. However, this is challenging because MCI does not cause specific individual symptoms.

Some symptoms of MCI have been reported recently. Early symptoms include executive function disorders [8,9,10,11], working memory disorders [10,11], attention disorders [9,11,12], visuo-constructional function disorders [13], and decreased upper extremity fine motor function [14,15,16]. Recent studies have revealed that MCI has various inconspicuous symptoms.

Several screening assessments for MCI are used worldwide. However, using conventional screening assessments to discriminate MCI has several limitations. Recently, the increase in cases of cognitive impairment progressing to dementia has led to rising concern and interest in diagnostic measures for neuropsychological deficiencies [17]. The Montreal Cognitive Assessment (MoCA) has a sensitivity of 80–100% and a specificity of 50–87% [18]. Meanwhile, the Mini-Mental State Examination (MMSE) has a sensitivity of 45–60% and a specificity of 65–90% [19,20,21]. Finally, the Trail Making Test Part B (TMT-B) has a sensitivity of 43% and a specificity of 91.1% [12]. These neuropsychological tests are comprehensive assessments that are useful in distinguishing MCI. However, some concerns have been raised regarding these methods of assessment. First, most people are unaware of the symptoms of MCI and do not undergo screening. Second, the examiners of neuropsychological tests require specialized knowledge. Third, neuropsychological testing can be exhausting for older adults owing to the long assessment times required [22]. Rapid and accurate screening and assessment of MCI are required to address the global dementia epidemic [23]. Therefore, screening for MCI should be (1) easy to perform in everyday life, (2) easy for anyone, and (3) less tiring for older adults.

In this study, we created a new screening test (the Cognitive Composition Test [CCT]) for MCI that can be performed anywhere and for anyone, and is associated with lower levels of fatigue. The CCT combines executive function, working memory function, attention, visuo-constructional function, and fine motor upper extremity function, all of which decline in patients with this condition, to examine the early symptoms of MCI.

The hypothesis of this study was that the CCT is effective in screening for MCI. In this study, we first examined the concurrent validity of the CCT and MoCA. Second, the HC and MCI groups were compared using the TMT-A, TMT-B, CCT-A, CCT-B, and CCT-C. Third, we calculated the prediction accuracy (sensitivity/specificity) and cutoff values.

## 2. Materials and Methods

### 2.1. Participants

The sample size was calculated using the G*power software (version 3.1.9.4) (Dusseldorf, Germany) [24] before starting the study. First, in the set of sample size calculations in the correlation coefficient analysis, “Correlation: Bivariate normal model” was selected. Under the “Type of power analysis” drop-down menu, “A priori: Compute required sample size-given α, power, and effect size” was selected. The “Tail” was set to 2; “Correlation ρ H1” was set to 0.3 (medium level); “α error probability” was set to 0.05; and “Power (1-β error probability)” was set to 0.8. The calculated sample size was 84. Second, for the set of unpaired t-tests, the difference between two independent means (two groups) was calculated. The type of power analysis selected was “A priori: Compute the required sample size, given α, power, and effect size”. The “Tail” was set to 2; “Effect size d” was set to 0.8 (large level); “α error probability” was set to 0.05; and “Power (1-β error probability)” was set to 0.95. The calculated sample size was 94.

Third, for the Mann–Whitney tests (two groups), the type of power analysis was “A priori: Compute the required sample size, given α, power, and effect size”. The “Tail” was set to 2; “Effect size” was set to 0.8, “α error probability” was set to 0.05, and “Power (1-β error probability)” was set to 0.95. The “Allocation ratio” was set to 2. The calculated sample size was 98 (sample size group, 1:33; group, 2:65).

A total of 112 participants were recruited for this study. However, 99 were finally included based on the exclusion criteria. They were classified into the healthy control (HC) and MCI groups. In this study, the criteria for the HC group were a MoCA score of 26 or higher [18] and an MMSE score between 28 and 30. Meanwhile, the criteria for the MCI group were a MoCA score of 25 or lower [18,25] and an MMSE score between 24 and 27 [26]. The classification results were as follows: 67 patients were in the HC group, 32 patients were in the MCI group, and 13 patients were excluded. The Edinburgh Handedness Inventory [27] was used to determine the dominant hand.

**Figure 1:**
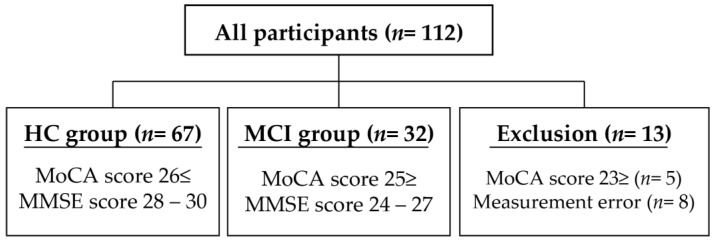
Classification of participants. HC Group—Healthy Control Group; MCI Group—Mild Cognitive Impairment Group; MoCA, Montreal Cognitive Assessment; MMSE, Mini-Mental State Examination.

The exclusion criteria excuded participants who (1) have diseases that would interfere with the study (dementia, visual impairment, and cerebrovascular disease) and (2) did not meet the mentioned criteria of the HC and MCI groups. Finally, measurement errors were calculated for TMT-A, TMT-B, CCT-A, CCT-B, and CCT-C (Figure 1, Table 1).

All participants were informed of the aim of the study and required to provide signed informed consent before participation. This study was approved by the Shijonawate Gakuen University Faculty of Rehabilitation Research Ethics Review Committee (approval no. 21-5) and conducted in accordance with the Declaration of Helsinki.

The HC group was divided into those with a MoCA score of 26 or higher and an MMSE score of 28–30, while the MCI group was divided into those with a MoCA score of 25 or lower and an MMSE score of 24–27. The measurement error is the measurement failure in the TMT-A, TMT-B, CCT-A, CCT-B, or CCT-C. Thirteen patients were excluded because they did not meet both of the criteria. 

### 2.2. Outcome Measures

This is a cross-sectional study. Information such as the age and sex of all participants were collected as baseline information, and handedness was collected using the Edinburgh handedness test. All participants were then tested in the following order: MoCA, MMSE, TMT-A, TMT-B, CCT-A, CCT-B, and CCT-C. After (1) MoCA, (2) MMSE, and (3) TMT, the participants were allowed to rest for 10 min to eliminate fatigue. Each neuropsychological examination was performed in a quiet single-occupancy room, and the examination was performed in the sitting position. In this study, each neuropsychological test was assessed for concurrent validity with the MoCA. The participants were then divided into the HC and MCI groups according to our criteria, based on their MoCA and MMSE scores. The performance times of TMT-A, TMT-B, CCT-A, CCT-B, and CCT-C were compared between the HC and MCI groups. Finally, the sensitivity, specificity, and cut-off values for the classification of the HC and MCI groups we calculated using each neuropsychological test.

#### 2.2.1. MoCA

Many clinicians and researchers routinely use MoCA to define MCI. The MoCA was developed as a cognitive screening tool to detect MCI in older adults. The cut-off score (less than 26 points) identifies MCI or dementia with high sensitivity (80–100%) and specificity (50–87%) [18].

In this study, we used the Japanese version of MoCA (MoCA-J). The MoCA and MoCA-J tests consist of assessments of visuospatial and executive functions, naming, memory, attention, recitation, word recall, abstract concepts, delayed recall, and disorientation [25].

#### 2.2.2. MMSE

MMSE scores, which reflect an individual’s global cognitive function, are clinically useful and have well-established utility [28,29,30]. The MMSE comprises items such as immediate and delayed word recall, sentence repetition, object naming, following a 3-stage command, and reading and writing a sentence, and the perfect score is 30 points [31]. A score of 23 or lower on the MMSE is indicative of dementia (sensitivity 81%, specificity 89%) [26]. Meanwhile, a score of 27 or lower is indicative of MCI (sensitivity 45–60%, specificity 65–90%) [19,20,21]. In this study, we used the Japanese version of MMSE (MMSE-J) [31,32].

#### 2.2.3. TMT-A, -B

TMT is widely used as a cognitive task to measure attention and executive function among older adults [33,34]. Two tasks were performed. In TMT Part A (TMT-A), consecutive numbers from 1 to 25 were connected using lines. In TMT Part B (TMT-B), similar lines were drawn, and numbers and letters were connected sequentially (e.g., 1-A–2-B). TMT-B is reported to be effective in discriminating between healthy older adults and those with MCI. Moreover, the sensitivity of the execution time was 43.0%, and the specificity was 91.1% [12]. The participants were instructed to draw a continuous line on the paper “quickly and accurately” to connect the stimuli. The procedures were based on the original version [35] and translated into Japanese (TMT-J) for this study (Example: 1-A–2-B, etc.), with reference to previous studies [36,37]. The TMT rating was defined as the performance time from start to finish for each task.

#### 2.2.4. CCT-A, -B, -C

**Figure 2:**
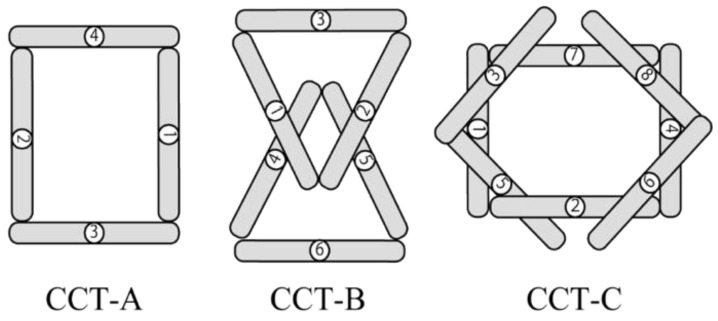
Three composition tests in CCT.

CCT involves creating the same diagram as the sample using sticks (width—11.4 cm, depth—1.0 cm, height—0.2 cm, weight—1.3 g) with numbers from ① to ⑧. Three types of CCT tasks were set according to the degree of difficulty. The difficulty level was set in terms of the number of sticks to be used and overlapping parts of the sticks based on the sample figures. The sample figures for CCT-A, CCTB, and CCT-C were set in order in the sample space. The participants were instructed to rearrange the top and bottom stacks of the sticks by referring to the sample figures. The simplest task, CCT-A, involved four sticks that did not overlap. Meanwhile, CCT-B involved six sticks and two overlaps. The most difficult task was CCT-C, which involved 8 sticks and 10 overlaps (Figure 2).

**Figure 3:**
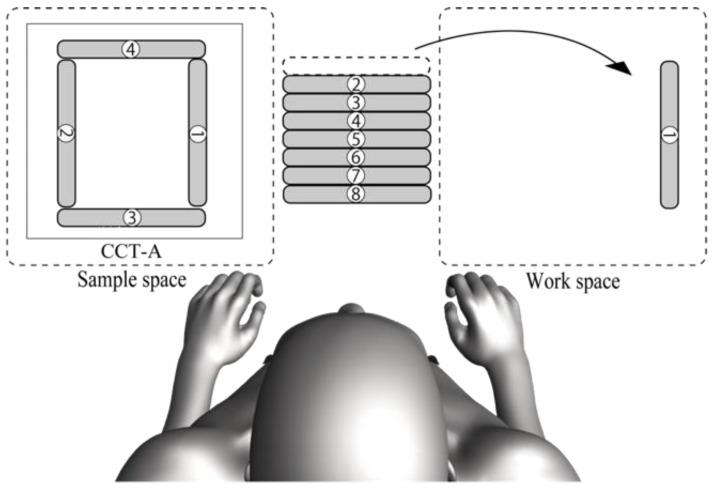
Experimental set-up for CCT-A.

In CCT, all participants were instructed to “(1) use the sticks in order starting with 1”, “(2) ensure that the numbers are in the same direction as the sample”, “(3) match the placement in the sample figure”, and “(4) make sure that the top and bottom of the stick are the same as the sample”. They were also instructed that they may use both hands for the task and perform the task as quickly as possible. As for the experimental set-up of the examination, the sticks to be arranged in numerical order were placed in the center; the sample figure was placed on the left side of the participant; and the workspace was placed on the right side (Figure 3). With the examiner’s cue, the participants were instructed to create the figures in numerical order. The sample figures were presented individually in the order of CCT-A, CCT-B, and CCT-C. The evaluation method measures performance time until the figure is completed. During CCT, the research data were excluded if the participant made one of the following mistakes: (1) the order of the sticks used was wrong; (2) the placement of the sticks was wrong; or (3) the vertical stacking of the sticks was in the wrong direction.

The CCT has the same composition as the composition task model, using sticks numbered ① to ⑧. There are three types of tasks, and the assessment calculates the sum of the performance times. CCT, Cognitive Composition Test.

The composition task model is placed on the left side. The sample figures for CCT-A, CCTB, and CCT-C are set in order in the sample space. The sticks labeled ① to ⑧ are placed in the center in the order of their numbers. The indicated workspace is on the right side of the participants. CCT, Cognitive Composition Test.

### 2.3. Statistical Analysis

The concurrent validity of MoCA and various neuropsychological tests (TMT-A, TMT-B, CCT-A, CCT-B, and CCT-C) was examined using Pearson’s correlation coefficient. The Shapiro–Wilk test was used to examine normality. For the HC and MCI groups, the performance times of TMT-A were analyzed using an unpaired t-test, and the performance times of TMT-B, CCT-A, CCT-B, and CCT-C were analyzed using the Mann–Whitney U test.

Receiver operating characteristic (ROC) curve analysis was performed for TMT-A, TMT-B, CCT-A, CCT-B, and CCT-C between the HC and MCI groups. The optimal cutoff value for the Youden index was defined as the point at which sensitivity + specificity −1 gives the highest value [38].

The statistical significance level was set at *p* < 5%. Statistical analyses were performed using IBM SPSS Statistics for Windows, version 28 (IBM Corp., Armonk, NY, USA).

## 3. Results

### 3.1. Concurrent Validity of MoCA and Each Neuropsychological Test

MoCA-J is the gold standard for testing MCI. The correlations between MoCA and TMT-A (r = 0.101, *p* = 0.321), TMT-B (r = −0.554, *p* = 0.000), CCT-A (r = −0.172, *p* = 0.088), CCT-B (r = −0.456, *p* = 0.000), and CCT-C (r = −0.713, *p* = 0.000) were examined.

### 3.2. Comparison of Each Neuropsychological Test between the HC and MCI Groups

**Figure 4:**
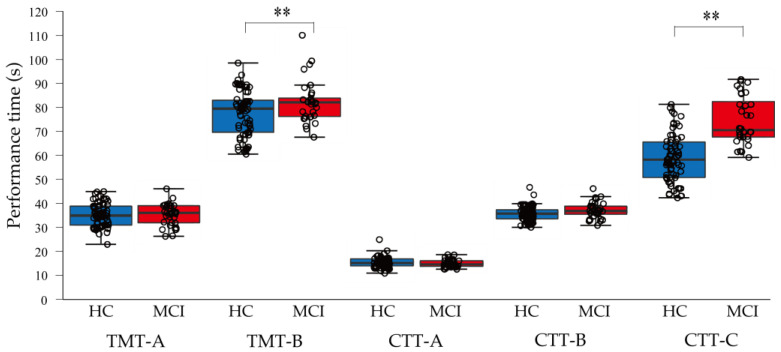
Comparison of each neuropsychological test between the HC and MCI groups. The vertical axis indicates the time (s) to complete each neuropsychological test. HC, healthy control; MCI, mild cognitive impairment; TMT-A, Trail Making Test Part A; TMT-B, Trail Making Test Part B; CCT, Cognitive Composition Test. **: *p* < 0.01.

In TMT-A, there was no significant difference between the HC (Ave ± SD: 35.58 ± 4.81 s) and MCI (35.5 ± 4.57 s, t = −0.002, *p* = 0.99, d = 4.73) groups. In TMT-B, the MCI group (median (25–75%): 82.4 (76.5–84.1) s) was significantly delayed compared to the HC group (80.2 (72.5–83.6), z = −2.09, *p* = 0.037). In CCT-A, there was no significant difference between the HC (median (25–75%): 14.6 (13.5–16.2) and MCI (14.6 (13.7–16.0), z = −0.007, *p* = 0.994) groups. In CCT-B, there was no significant difference between the HC (median (25–75%): 35.6 (33.5–38.0) and MCI (36.5 (35.2–38.5), z = −1.943, *p* = 0.052) groups. Furthermore, in CCT-C, the MCI group (median (25–75%): 70.6 (67.6–82.7)) was significantly delayed compared to the HC group (62.6 (55.5–71.3), z = −5.881, *p* = 0.001) (Figure 4).

### 3.3. Discrimination Sensitivity and Cutoff Value of CCT-C

**Figure 5:**
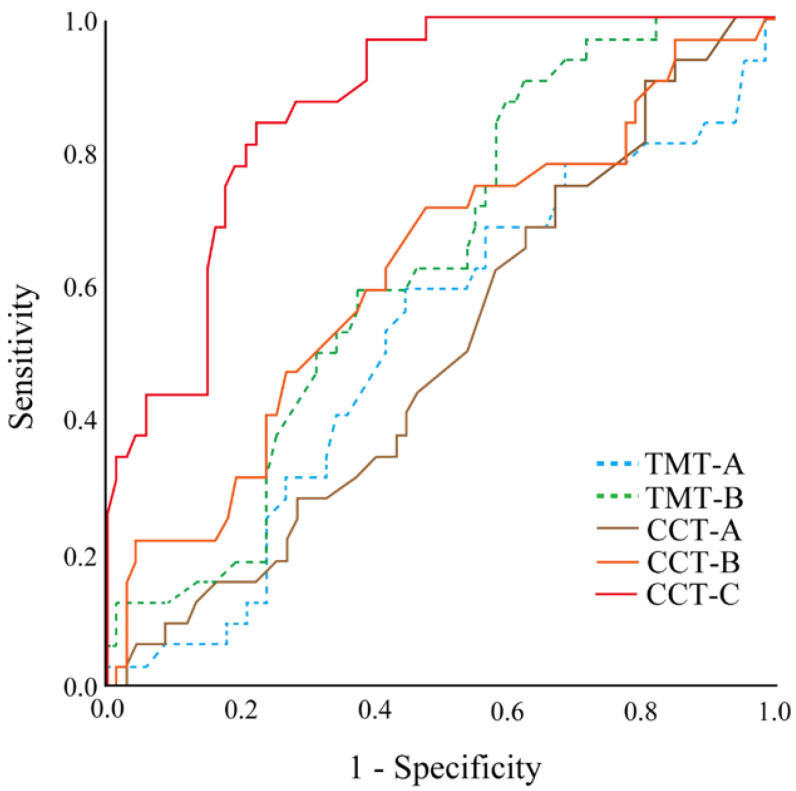
Receiver operating characteristic (ROC) curves of each neuropsychological test. ROC curves predicting HC or MCI with the TMT-A, TMT-B, CCT-A, CCT-B, and CCT-C (time). HC, healthy control; MCI, mild cognitive impairment; TMT-A, Trail Making Test Part A; TMT-B, Trail Making Test Part B; CCT, Cognitive Composition Test.

The area under the ROC curve (AUC) of TMT-A was 0.513 (*p* = 0.840), and the optimal cut-off value was 35.70 s (sensitivity: 59%, specificity: 55%). The AUC of TMT-B was 0.630 (*p* = 0.037), and the optimal cut-off was 73.55 s (sensitivity: 91%, specificity: 37%). The AUC of CCT-A was 0.500 (*p* = 0.994), and the optimal cut-off was 13.05 s (sensitivity: 91%, specificity: 19%). The AUC of CCT-B was 0.621 (*p* = 0.052), and the optimal cut-off was 35.55 s (sensitivity: 72%, specificity: 52%). The AUC of CCT-C was 0.867 (*p* = 0.000), and the optimal cut-off was 65.75 s (sensitivity, 84%; specificity, 78%) (Figure 5 and Table 2).

## 4. Discussion

### 4.1. Effectiveness of CCT

We developed the CCT, which can be easily used to screen for MCI. In the CCT, a number of components—executive function, working memory function, attention, structural function, and declining upper extremity function—were combined and considered as early symptoms of MCI. This study hypothesized that the CCT, which was created from a combination of early MCI symptoms, would be an effective screening tool for the early detection of MCI. First, we examined the concurrent validity of MoCA, which was the gold standard for screening MCI, and found that TMT-B, CCT-B, and CCT-C were negatively correlated with MoCA. Subsequently, a comparison of the results for TMT-A, TMT-B, CCT-A, CCT-B, and CCT-C between the HC and MCI groups showed that the MCI group was significantly delayed in TMT-B and CCT-C compared with the HC group. Finally, we examined the predictive accuracy (sensitivity/specificity) of the discrimination accuracy between the HC and MCI groups and found that CCT-C had a higher discrimination ability than TMT-A, TMT-B, CCT-A, and CCT-B. CCT-C is therefore an effective screening tool for identifying MCI.

CCT-B and CCT-C have significant negative correlations with MoCA. CCT assignments include elements of TMT. The components required for TMT-A are visual searching, visual attention, and motor speed [39,40]. TMT-A is a simple and less mentally tiring test. TMT-B includes the elements of TMT-A, but requires verbal intelligence, the ability to mentally maintain two simultaneous sequences, memory, and cognitive alternation [39,41]. In other words, TMT-B contains more elements than TMT-A; thus, it is more difficult. The CCT is a screening test created to include the elements of TMT-B and also executive function, working memory function, attention, visuo-constructional function, and fine motor upper extremity function. CCT-A, CCT-B, and CCT-C differed in terms of difficulty and performance time. We believe that the CCT-C task showed a correlation with MoCA because both the time spent on the task and the difficulty level were effective in discriminating MCI.

Second, TMT-B and CCT-C may be useful in discriminating between the HC and MCI groups. In this study, patients were divided into the HC and MCI groups based on the MoCA and MMSE criteria [18,25,26]. For TMT-B, the HC group had a median of 80.2 (25–75%: 72.5–83.6), and the MCI group had a median of 82.4 (76.5–84.1), with a significant difference (*p* = 0.037). In CCT-B, the HC group had a median of 35.6 (33.5–38.0), and the MCI group had a median of 36.5 (35.2–38.5). The MCI group had a delay in performance time close to the significance level (*p* = 0.052). In CCT-C, the HC group had 62.6 (55.5–71.3), and the MCI group had 70.6 (67.6–82.7), with a significant delay in the MCI group (*p* = 0.001). In this study, the difficulty of the CCT was set at three levels. We concluded that CCT-C, which had the highest difficulty, was effective in discriminating MCI. The difficulty levels of TMT-B and CCT-C may be useful in discriminating between the HC and MCI groups.

Third, the CCT-C was the most effective, in terms of screening ability, in discriminating between the HC and MCI groups. TMT-A was not effective in discriminating MCI. Meanwhile, TMT-B had a good discrimination sensitivity (sensitivity 43.0%, specificity 91.1%) between healthy older adults and those with MCI, indicating its effectiveness [12]. Therefore, TMT-A does not have a valid discrimination accuracy. TMT-B had an AUC of 0.630 and discriminative accuracy with an optimal cut-off value of 73.55 s (sensitivity 90.6%, specificity 37.3%). CCT-C had an AUC of 0.8667 with an optimal cutoff value of 65.75 s (sensitivity 84.4%, specificity 77.6%). It has a “moderately accurate” level of discrimination accuracy, with an AUC value of 0.7–0.9 [42]. In other words, although this was at a moderate level, the accuracy of the CCT was still considered to be high. Nevertheless, the CCT-C was considered to be the screening method with the highest discrimination accuracy compared with TMT-B.

The findings of this study indicate that the CCT-C, as a screening test, has concurrent validity with MoCA and can discriminate between patients with MCI and healthy participants. It can be performed anywhere and for anyone, with lower levels of associated fatigue. MCI does not have noticeable symptoms; however, the CCT can easily evaluate and detect it during the early stages. The CCT is an effective way to monitor cognitive status in resource-poor settings. In this regard, CCT is easier to use than previous neuropsychological tests. Furthermore, this study was a multicenter study and supports the efficacy of the CCT. Thus, we believe that the CCT can serve as a countermeasure to the increase in dementia, which has become a global social phenomenon.

Nevertheless, future research may focus on considering ways to further reduce the burden on patients and improve the discrimination accuracy of the CCT-C test.

### 4.2. Limitations

One limitation of this study was that MoCA and MMSE scores determined the selection criteria for the MCI group. Therefore, the MCI group were classified without a diagnosis. Second, the participants’ educational backgrounds were not collected as basic information. Third, the degree of fatigue was only considered in relation to the performance time of the test. Fourth, the method used in this study could not distinguish among MCI types.

## 5. Conclusions

The CCT can effectively be used for screening, even during the MCI stage when symptoms are difficult to observe. Moreover, its sensitivity for discrimination was higher than that of TMT-B. In addition, the test can be performed quickly, anywhere, with less fatigue associated, and by anyone, even those with no knowledge of neuropsychology.

## Figures and Tables

**Table 1:** Demographic characteristics of the participants.

	HC (Mean ± SD)	MCI (Mean ± SD)	*p*-Value	Cohen’s d
Age	72.7 ± 4.8	73.9 ± 4.7	0.22	0.216
Sex (male, female)	M: 27 F:40	M: 14 F: 18	-	-
Dominant hand ^1^	R: 61 L: 6	R: 30 L: 2	-	-
MoCA	27.4 ± 1.2	23.2 ± 1.42	0.001	0.359
MMSE	28.6 ± 0.8	25.5 ± 1.19	0.001	0.365

^1^ Dominant hand was assessed using the Edinburgh handedness inventory. HC, healthy control; MCI, mild cognitive impairment; MoCA, Montreal Cognitive Assessment; MMSE, Mini-Mental State Examination.

**Table 2:** ROC curves of each neuropsychological test.

	AUC	*p*-Value	Cut-Off	Sensitivity	Specificity
TMT-A	0.513	0.840	35.70	59.4%	55.2%
TMT-B	0.630	0.037	73.55	90.6%	37.3%
CCT-A	0.500	0.994	13.05	90.6%	19.4%
CCT-B	0.621	0.052	35.55	71.9%	52.2%
CCT-C	0.867	0.000	65.75	84.4%	77.6%

## Data Availability

The data presented in this study are openly available in Mendeley Data at https://doi.org/10.17632/gx75cp3g4z.1, reference number [43].
